# A signal-on built in-marker electrochemical aptasensor for human prostate-specific antigen based on a hairbrush-like gold nanostructure

**DOI:** 10.1038/s41598-017-11680-5

**Published:** 2017-09-11

**Authors:** Naghmeh Sattarahmady, Amid Rahi, Hossein Heli

**Affiliations:** 1 Nanomedicine and Nanobiology Research Center, Shiraz University of Medical Sciences, Shiraz, Iran; 2 Department of Medical Physics, School of Medicine, Shiraz University of Medical Sciences, Shiraz, Iran; 3 Student Research Committee, School of Medicine, Shahid Beheshti University of Medical Sciences, Tehran, Iran

## Abstract

A green electrodeposition method was firstly employed for the synthesis of round hairbrush-like gold nanostructure in the presence of cadaverine as a size and shape directing additive. The nanostructure which comprised of arrays of nanospindles was then applied as a transducer to fabricate a signal-on built in-marker electrochemical aptasensor for the detection of human prostate-specific antigen (PSA). The aptasensor detected PSA with a linear concentration range of 0.125 to 128 ng mL^−1^ and a limit of detection of 50 pg mL^−1^. The aptasensor was then successfully applied to detect PSA in the blood serum samples of healthy and patient persons.

## Introduction

Prostate cancer (PCa) is the most common cancer in men and is the second-leading cause of cancer mortality in men. This cancer accounts for sixth percent of the total cancer deaths in males^1^. Therefore, early diagnosis is very important to prevent PCa.

Prostate-specific antigen (PSA) is a biomarker that is most widely employed for the detection of PCa^2^. It has been shown that PSA is the most validated biomarker for the early detection of prostate cancer and monitoring the disease recurrence after treatment^3^. PSA is a glycoprotein with chymotrypsin-like protease activity and exists naturally in the human serum, either free or in combination with various proteinase inhibitors^4^. Increment in the PSA concentration to >4.0 ng mL^−1^ is usually suspected to the appearance of tumors in the prostate and a biopsy should be performed^5^. A total PSA level higher than 10 ng mL^−1^ is generally considered with PCa^6^. Therefore, the development of reliable, specific, rapid and simple methods for determination of PSA is very important in the early diagnosis of PCa or monitoring of the disease after treatment.

Up to now, different methods have been reported for the detection of PSA including radioimmunoassay^7^, fluorescence detection^8^, enzyme-linked immunosorbent assay^9^, surface plasmon resonance^10^, chemiluminescent immunoassay^11^, surface-enhanced Raman scattering^12^, and electrochemical methods^13^. Some of these methods employ antibody assays which are expensive, inactive, inconvenient, time-consuming and complicated, and suffer from the disadvantages of antibodies related to the production, stability, and manipulation^14, 15^. In addition, biosensors based on PSA aptamers with optical^16, 17^ or electrochemical^5, 18–20^ transductions have been reported. Aptamers, as artificial single-stranded DNA or RNA, selectively bind to small biomolecules and complex species such as cells^21^. Aptamers are one of the suitable choices for recognition due to their advantages including high binding affinity, good stability, high specificity, and wide range of detection^22^. However, already reported electrochemical PSA aptasensors suffered from the drawbacks of complexity of fabrication^5, 19^, multiple materials and binding steps needed for immobilization of aptamers^5, 18^, or high cost of the detection devices^20^. Therefore, development of novel biosensors for PSA detection is highly interested. Besides, electrochemical methods have advantages. Some of them are ease of miniaturization, high sensitivity, low cost, and rapid response^23, 24^.

Nanotechnology plays a principal role in the fabrication of electrochemical sensors and biosensors^25–31^. Modification of the electrode surface by novel nanomaterials is an effective step to improve the performance of electrochemical aptasensors^32^. Due to the advantages of gold nanostructures including high surface area and electron transfer rate, and unique chemical characteristics, they are promising nanomaterials for immobilization of aptamers^33, 34^. In addition, gold nanostructures are being used to improve the selectivity and sensitivity of detection^28, 33–37^. A combination of aptamers with nanomaterials to fabricate electrochemical PSA biosensors provide sensitive and specific methods of PSA determination^5, 34, 38^. In the present study a built in-marker aptamer was employed for PSA detection based on a transducer of hairbrush-like gold nanostructure.

**Figure 1:**
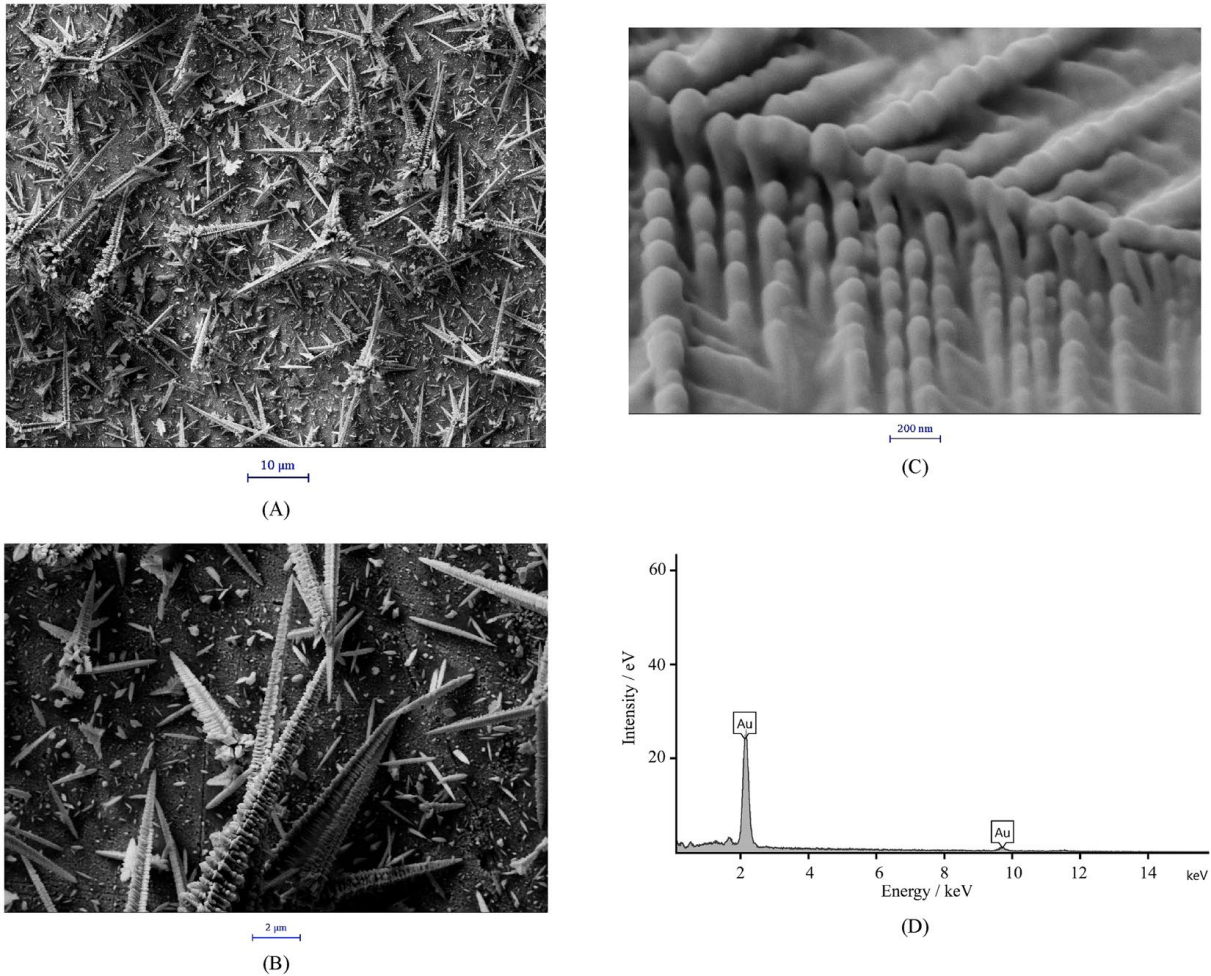
FESEM images with different magnifications (**A**–**C**) and an EDS spectrum (**D**) of the HL-Au electrode surface.

Figure 1 presents FESEM images (A-C) of the HL-Au electrode surface with different magnifications. Low-magnified images indicate that the surface was covered by round hairbrush-like rods. High-magnified image indicates that the rods surface comprised of spindles of 30–150 nm in diameter. Figure 1D shows an EDS spectrum of the hairbrush-like gold nanostructure confirming that the deposited layer comprised of pure gold. The shape and size of the hairbrush-like gold nanostructure provides an appropriate substrate (as a transduction element) for aptamer immobilization, and can promote aptamer-PSA binding^39^. Real surface area of the hairbrush-like gold nanostructure was measured, and it was obtained that the surface area of the HL-Au electrode is ∼1.6 times of the Au electrode. Therefore, the HL-Au electrode has a larger surface area providing a high surface concentration of immobilized aptamer for a higher sensitive biosensing.

For electrodeposition of the hairbrush-like gold structure a negative overpotential of 0 V (vs. AgCl) was applied. This leads to a fast gold atom formation^40^ and producing many randomly distributed nuclei at the surface. On the other hand, cadaverine is simultaneously adsorbed on the (1 1 1) plane of the nuclei^41^. This is due to the binding affinity of polyamines (such as cadaverine) to the gold surface^42–44^. Cadaverine also provides a positive charge surface due to its protonation in the synthesis solution, and therefore, more adsorption of AuCl_4_^−^ ions on the (1 1 1) plane occurs following via preferential growth along the (1 1 1) directions by the diffusion-limited aggregation mechanism^45^.

**Figure 2:**
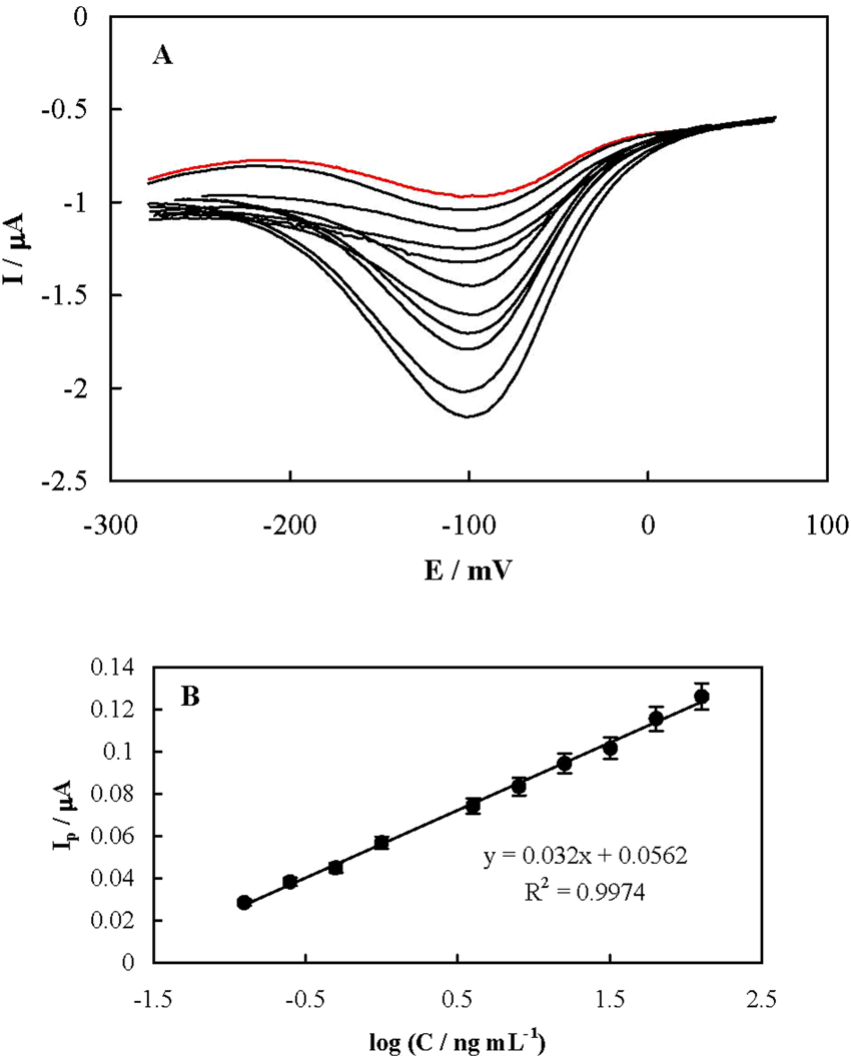
(**A**) DPVs recorded using the aptasensor in Tris before (red line) and after (black lines) binding with different concentrations of PSA of 0.125, 0.25, 0.5, 1.0, 2.0, 4.0, 8.0, 16, 32, 64 and 128 ng mL^−1^. The arrow shows the direction of PSA concentration increment. (**B**) The dependency of the peak current on the PSA concentration (the calibration curve).

DPVs recorded for the aptasensor in Tris before and after binding with different concentrations of PSA are shown in Fig. 2A. PSA binding induces aptamer folding into a new structure decreasing the distance between MB and the aptasensor surface. Therefore, the electron transfer efficiency is accelerated. Figure 2B shows the dependency of the DPVs peak currents on the PSA concentration. This calibration plot is linear in the range of 0.125 to 128 ng mL^−1^ of PSA with a regression equation of y = −(0.032 ± 0.0006)x + (0.0562 ± 0.0007). Based on this plot, a limit of detection (LOD, 3δ/m) of 0.04 ng mL^−1^ for PSA was attained. In Table 1, a comparison between LOD values reported for the PSA detection methods is summarized.

**Table 1:** A comparison between the PSA detection methods.

Detection method	Recognition element	Linear range (ng mL^−1^)	LOD (pg mL^−1^)	Reference
Fluorescence	Dye-labeled aptamer/MoS_2_ nanosheets	0.5–300	200	16
Chemiluminescence	Dye-labeled aptamer/Fe_3_O_4_-graphene oxide nanoparticles	1.6–50	500	17
Electrochemistry	Aminated aptamer	1–100	1000	18
Electrochemistry	Biotinated aptamer	0.25–200	250	5
Electrochemistry	Aptamer-MIP hybrid	0.1–100	1	19
Electrochemical impedance	Aptamer	0.007–6	1000	20
Electrochemistry	Aptamer	0.125–200	50	34
Surface acoustic wave detection	Aptamer beacon	10–1000	1.0 × 10^4^	50
RLS assay	Aptamer-modified AuNPs	0.13–110	320	51
Electrochemical immunoassay	Label-free PSA antigen	0.05–5	13	52
Electrochemical immunoassay	Graphene/cobalt hexacyanoferrate	0.02–2	100	53
Electrochemical immunoassay	Magnetic beads enzyme linked immunosorbent	0–1	<100	54
Voltammetric ELISA	DAB-H_2_O_2_-HRP	0.2–32	100	55
Electrochemical immunoassay	Ab_1_-Ag-Ab_2_-HRP	0–15	250	56
Electrochemical dual sensing	Enzyme/antibody	1–10	1000	57
Colorimetric assay	PSA peptide	0.1–100	1.0 × 10^4^	58
Optical	Antibody	0.0005–5	10	59
Duplexed immunoassay	Functional microbeads	0.01–5	50	60
Cathodic ECL immunoassay	Antibody/luminal/graphene	0.01–8	8	61
Sandwich-type immunoassay	Antibody on PDMS microfluidic chips	4–10	520	62
Chemiluminescence	Dye-labeled aptamer	1.9–125	1.0 × 10^3^	63
SIA	Antibody	0.001–1000	0.11	64
ECL	Sandwich-type immunoreaction	0.001–10	0.3	65
Electrochemistry	Graphene oxide/ssDNA/PLLA nanoparticles	0.05–100	1.0 × 10^3^	66
Bridge-shaped PZT resonator	Antibody	0.01–0.1	4.0 × 10^3^	67
Magnetic immunoassay	SERS-based microdroplet	174 droplets per minute	100	68
Electrochemistry	Peptide cleavage	0.001–30	0.78	69
Imprinted capacitive biosensor	Microcontact-PSA-MIP	0.00001–100	0.6	70
Microarray immunoassay	Antibody	0.005–500	5	71
Electrochemistry	Aptamer-attached marker (MB)	0.125–128	40	This work

Abbreviations: Ab_1_-Ag-Ab_2_-HRP: Antibody1-antigene-antibody 2- horseradish peroxidase. AuNPs: Gold nanoparticles. DAB-H_2_O_2_-HRP: 3,3′-diaminobenzidine-H_2_O_2_-horseradish peroxidase. ECL: Electrochemiluminescence. LOD: Limit of detection. MB: Methylene blue. MIP: Molecularly imprinted polymer. PDMS: Poly(dimethylsiloxane). PLLA: Poly-L-lactide. PZT: Lead zirconate titanate. RLS: Resonance light scattering. SIA: Surface-enhanced Raman scattering (SERS)-based immunoassays. ssDNA: Single stranded DNA.

**Figure 3:**
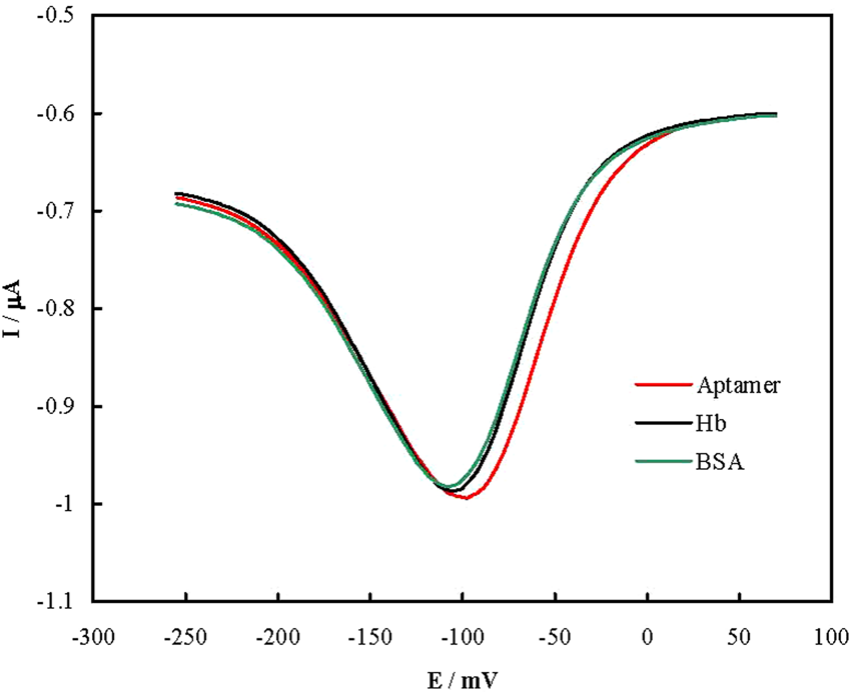
DPVs recorded using the aptasensor in Tris before and after binding with 50 mg mL^−1^ bovine serum albumin (green) or 17.5 mg mL^−1^ hemoglobin (black).

In order to inspection the selectivity of the aptasensor, DPVs for binding the non-specific proteins of hemoglobin and bovine serum albumin were recorded and shown in Fig. 3. The peak currents had a little decrement. It should also be noticed that the blood serum carries different biologicals such as enzymes, proteins, hormones and antibodies with high concentrations. The aptasensor could directly analyze PSA in the serum samples with small values of constant error, and therefore, it can be considered as selective.

In order to apply the aptasensor for PSA detection in real samples, blood serum samples of healthy and patient persons (already confirmed by an immunoradiometric assay) were analyzed. It was obtained that peak currents in DPVs recorded for the healthy samples were near the same as that for the absence of PSA (data not shown). The PSA levels of the patient samples could be quantified, and compared with those obtained by the immunoradiometric assay in Table 2. The results indicated that the aptasensor can be applied for PSA detection in patients and clinical diagnosis.

**Table 2:** Values of the PSA concentrations in the patients’ blood serum obtained by the aptasensor.

Nominal value^a^ (ng mL^−1^)	Obtained value (ng mL^−1^)	Bias (%)
5.31	5.2	−2.1
6.78	6.94	2.4
6.87	6.99	1.7
8.48	8.54	0.7
8.71	8.72	0.1
9.19	8.95	−2.6
10.92	11.01	0.8
12.31	11.88	−3.5
13.31	13.60	2.2
19.00	18.46	−2.8

^a^Nominal values were obtained by the immunoradiometric assay.

**Figure 4:**
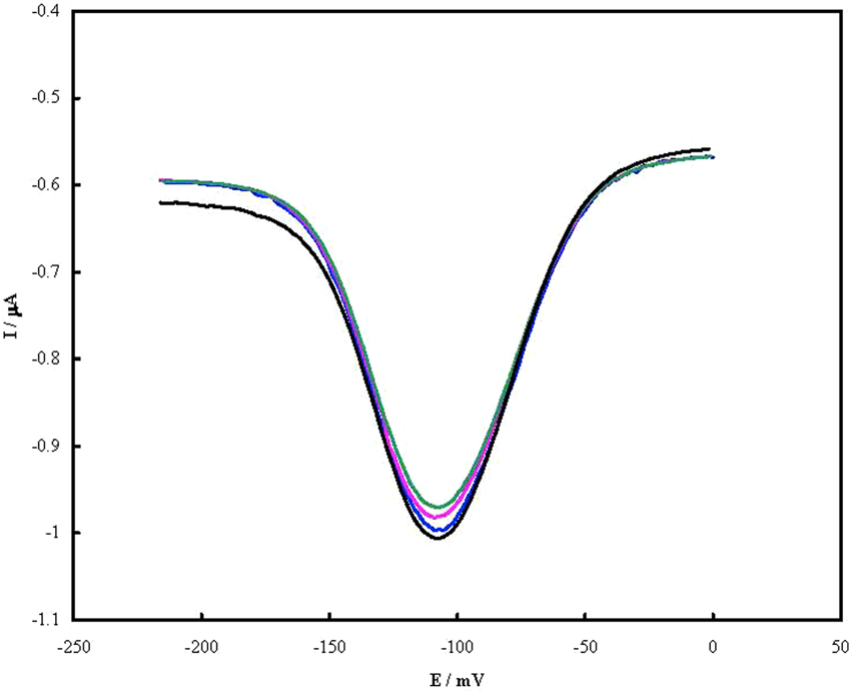
DPVs of the aptasensor in Tris before for the repeating fabrication.

Reproducibility of fabrication of the aptasensor was inspected by immersion in the Piranha solution for 30 s and re-fabrication (n = 4), and DPVs were recorded (Fig. 4). The results showed a relative standard deviation (RSD) of 3.8% for the peak current.

**Figure 5:**
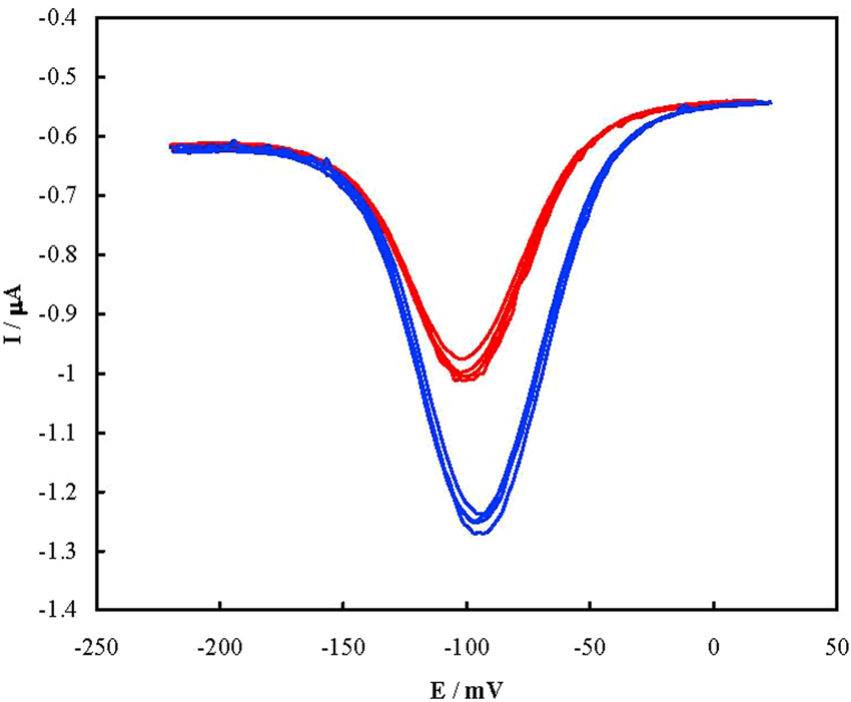
DPVs of the aptasensor in Tris before (red curves) and after (blue curves) reparative binding with 1.0 ng mL^−1^ PSA.

In order to inspect the regeneration of the aptasensor, it bonded with PSA for 1 h at 37 °C and then immersed in hot water of 70 °C for 5 min to release PSA. Then it was cooled down slowly to room temperature and re-bonded with the same PSA concentration (n = 4). DPVs were recorded after repeated binding with 1.0 ng mL^−1^ PSA and shown in Fig. 5. Based on the data, a RSD of 4.1% was obtained for regeneration of the aptasensor.

To evaluate the stability of the aptasensor, DPVs were recorded for 1.0 ng mL^−1^ PSA in consecutive days, and aptasensor was stored in Tris at 4 °C. The peak current did not regularly change at least for 25 days.

In summary, hairbrush-like gold nanostructure was firstly electrodeposited in the presence of cadaverine. It seems that the molecules bearing multiple amine functional groups can enforce gold nucleation and growth process in a special direction, and produce special shape and size of gold nanostructures. A specific aptamer for PSA was then modified with MB (as a marker) to produce a built in-marker aptamer. The gold nanostructure, as a transducer, was employed for immobilization of a high surface concentration of the aptamer and fabrication of an aptasensor. Aptasensor was signal-on type, had a low LOD for PSA, can detect PSA in patients’ samples, and would be applicable for clinical analysis.

## Methods

### Materials

All chemicals were of analytical grade from Scharlau (Spain) or Merck (Germany). All solutions were prepared by redistilled water. A specific aptamer sequence for PSA which was modified with methylene blue (MB) was employed with following sequence. It has been reported that this sequence has a high affinity to PSA^46^ and modified with MB in this study: 5′ SH-(CH_2_)_6_ TT TT TA AT TA AA GC TC GC CA TC AA AT AG CT TT-3′-MB.

The aptamer was purchased from Bioneer (Korea). PSA, hemoglobin and bovine serum albumin were purchased form Sigma (USA). The aptamer stock solutions were prepared with a 20 mmol dm^−3^ Tris-HCl buffer, pH 7.4 solution (Tris) and kept frozen.

### Apparatus

Electrochemical measurements were performed in a three-electrode cell connected to a μ-Autolab potentiostat/galvanostat (the Netherlands). An Ag/AgCl, 3 mol dm^−3^ KCl, a platinum rod, and a gold disk (Au, 2 mm of diameter) or the Au electrode deposited with hairbrush-like gold nanostructure (HL-Au electrode) were employed as the reference, counter and working electrodes, respectively. The system was run on a PC by GPES 4.9 software.

Field emission scanning electron microscopy (FESEM) was performed using a Zeiss, Sigma-IGMA/VP (Germany) equipped with energy-dispersive X-ray spectroscopy (EDS). The samples were coated by a 2–5 nm thin film of gold by sputtering.

### Preparation of HL-Au electrode

Firstly, the Au electrode was polished on a polishing pad with 0.05 μm-alumina powder lubricated by water. Polishing was continued to attain a mirror-like surface. The electrode was then cleaned by immersion in a 1:3 water/ethanol mixture and ultrasonication for 8 min in an ultrasound bath. It was further electropolished in a 500 mmol dm^−3^ H_2_SO_4_ solution and applying cyclic potential in the range of cathodic to anodic edges of the electrolyte stability for 20 consecutive cycles. The Au electrode was then transferred to a cell containing the electrodeposition solutions containing 20 mmol dm^−3^ HAuCl_4_ + 500 mmol dm^−3^ H_2_SO_4_ + 150 mmol dm^−3^ cadaverine. Electrodeposition was done at 0 mV for 600 s. The nsAu electrode was then rinsed thoroughly with distilled water.

### Immobilization of the aptamer

Lyophilized aptamer was dissolved in distilled water. Then, 10 µL dithiothreitol (DTT) solution (containing 10 mmol dm^−3^ sodium acetate, pH 5.2 and 500 mmol dm^−3^ DTT) was added, mixed, and incubated at room temperature for 15 min. Excess DTT and thiol fragments were removed from the mixture by extraction with ethyl acetate (three times, total volume of 150 µL), and the upper layer was discarded, the next step was then immediately performed. Immobilization of aptamer was done by dropping 10 μL of 10.0 μmol dm^−3^ aptamer solution dissolved in Tris on the HL-Au electrode surface and kept at 4 °C for 8 h^11, 28, 34^. Then, the electrode was rinsed with Tris, and further treated with 1.0 mmol dm^−3^ 6-mercapto-1-hexanol at room temperature for 30 min to obtain a well aligned aptamer monolayer^11, 28, 34^. Then, the electrode was washed again with Tris and double distilled water, respectively, to remove non-specific adsorbed thiols. The obtained electrode was denoted as aptasensor

### PSA binding

PSA binding process was performed by immersing the aptasensor into Tris containing various concentrations of PSA for 1 h at 37 °C^47, 48^ and then rinsed with Tris.

### Electrochemical measurements

The real surface areas of the Au and HL-Au electrodes were electrochemically determined. The electrodes were transferred to a solution of 0.5 mol dm^−3^ KCl containing 0.5 mmol dm^−3^ K_4_[Fe(CN)_6_], and cyclic voltammograms at different potential sweep rates were recorded. Using the Randles-Sevcik equation^25^ and the value of 7.60 × 10^−6^ cm s^−1^ for the diffusion coefficient of [Fe(CN)_6_]^4−^ ^49^, the real surface areas of the Au and HL-Au electrodes were measured.

Electrochemical detection of PSA was done in an electrochemical cell containing 10 mL Tris by recording differential pulse voltammograms (DPVs) for the reduction peak of aptamer built in-MB. DPVs were recorded with a pulse width of 25 mV, a pulse time of 50 ms, and a scan rate of 10 mV s^−1^. All electrochemical measurements were performed at room temperature.

### Human samples

Blood samples of patient and healthy persons in our study provided by Faghihi Hospital Shiraz (Iran), and approved by Ethics Committee of Shiraz University of Medical Sciences (Number 12292). All methods were performed in accordance with the approved guidelines, and the institutional review board waived the need to informed consent for patient samples. The samples were taken by laboratory of the hospital and were kept until certain time. All collected samples were kept frozen until assay. The PSA levels of the samples were firstly determined by an immunoradiometric assay, and then, were diluted with distilled water (1:9) and assayed by aptasensor.
